# FIMTrack: An open source tracking and locomotion analysis software for small animals

**DOI:** 10.1371/journal.pcbi.1005530

**Published:** 2017-05-11

**Authors:** Benjamin Risse, Dimitri Berh, Nils Otto, Christian Klämbt, Xiaoyi Jiang

**Affiliations:** 1 School of Informatics, University of Edinburgh, Edinburgh, Scotland, United Kingdom; 2 Faculty of Mathematics and Computer Science, University of Münster, Münster, Germany; 3 Department of Behaviour and Neuronal Biology, University of Münster, Münster, Germany; Universite de Montreal, CANADA

## Abstract

Imaging and analyzing the locomotion behavior of small animals such as Drosophila larvae or C. elegans worms has become an integral subject of biological research. In the past we have introduced FIM, a novel imaging system feasible to extract high contrast images. This system in combination with the associated tracking software FIMTrack is already used by many groups all over the world. However, so far there has not been an in-depth discussion of the technical aspects. Here we elaborate on the implementation details of FIMTrack and give an in-depth explanation of the used algorithms. Among others, the software offers several tracking strategies to cover a wide range of different model organisms, locomotion types, and camera properties. Furthermore, the software facilitates stimuli-based analysis in combination with built-in manual tracking and correction functionalities. All features are integrated in an easy-to-use graphical user interface. To demonstrate the potential of FIMTrack we provide an evaluation of its accuracy using manually labeled data. The source code is available under the GNU GPLv3 at https://github.com/i-git/FIMTrack and pre-compiled binaries for Windows and Mac are available at http://fim.uni-muenster.de.

This is a *PLOS Computational Biology* Software paper.

## Introduction

For most animals the ability to move is essential to survive. A complex nervous system, built-up by neurons and glial cells, allows sophisticated locomotion control. The analysis of locomotion of freely moving animals is crucial to gather insights into the nervous system functionality. Especially *Drosophila melanogaster* larvae and *Caenorhabditis elegans* worms are popular model organisms in neuro- and behavioral biology since sophisticated genetic tools and a well-established knowledge base provide advantages like cell specific manipulations and ease behavioral inferences [1, 2]. Different tracking and locomotion analysis tools have been proposed including commercially available (e.g. EthoVision [3]) and custom solutions (e.g. MWT [4], MAGAT [5], SOS [6]). In the past we have introduced a novel imaging technique called FIM [7] to gather high-contrast recordings of the aforementioned model organisms. The associated open-source tracking software FIMTrack has already been used in a variety of studies [7–11] and a video tutorial has been published in [12] to demonstrate its biological usability. For example, FIMTrack has successfully been used to identify a central neural pathway for odor tracking in Drosophila [9] and to study the behavioral changes of *uba-5* knockout C. elegans worms [13].

Here we elaborate on the technical aspects and algorithms implemented in FIMTrack for a better understanding of the resultant quantities. Additionally, we provide an accuracy quantification using manually labeled data.

FIMTrack offers several advantages compared to state-of-the-art tracking tools:

- The assignment of animals across frames is implemented in a modular fashion, offering different combinations of assignment strategies and cost functions, making FIMTrack more flexible for a wider range of model organisms, locomotion types, and camera properties.
- FIMTrack extracts a huge variety of posture and motion-related features with a very high tracking accuracy which is evaluated using labeled data.
- Our tracking program has an intuitive graphical user interface allowing the inspection of most of the calculated features, an option for manual tracking, and an easy integration of stimulus regions.
- FIMTrack does not rely on commercial packages and is available in source code and as pre-compiled binaries for Windows and Mac.
- The software is implemented in an object-oriented fashion to improve re-usability and enable extensibility.

The main purposes of this paper are:

- Elaborate the algorithmic insights of the widely used FIMTrack software to enable easier usage and extensibility.
- Provide a ground truth-based evaluation of the tracking performance.
- Give an update on the current state of the program featuring a variety of novel functionality compared to its first usage in 2013 [7].
- Introduce FIMTrack as a tool for other communities dealing with other model organisms.

## Design and implementation

FIMTrack is written in C++ and is easily extendable since the object-oriented programming paradigm is used. We utilize the OpenCV library and the Qt framework in combination with QCustomPlot (http://qcustomplot.com/) for image processing and the graphical user interface. Generally, FIMTrack consists of three main modules, namely the *tracker*, the *results viewer*, and the *input-output (IO)* module.

### Tracker module

**Fig 1:**
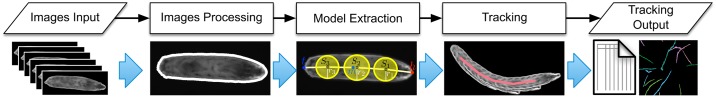
Flow chart of the tracking module.

The main flow of the tracking module is given in Fig 1 and can be separated into *image processing*, *model extraction*, and *tracking*.

#### Image processing

Let $I_{t}$ be the gray scale image at time *t* and assume that *N* animals in total need to be tracked. Prior to further image analysis we compute a static background image which includes almost all immovable artifacts. Since images produced by FIM have a black background with bright foreground pixels and since we assume that an animal moves more than its own body length during the recording, the calculation of the background image B can be done using the minimal pixel intensity value over time, resulting in

$$B\left(r,c\right)=\min_{t}\left(I_{t}\left(r,c\right)\right)$$

where $I_{t}\left(r,c\right)$ is the pixel intensity at row *r* and column *c* at time *t*.

Subsequently, the foreground image $F_{t}$ containing almost all objects of interest without the artifacts present in the background image B is obtained by

$$F_{t}\left(r,c\right)=\left\{\begin{array}{cc} I_{t}\left(r,c\right) & \text{if}\;|I_{t}\left(r,c\right)-B\left(r,c\right)|\geq \tau_{B}\left(r,c\right)\\ 0 & \text{otherwise} \end{array}\right.$$

with $\tau_{B}\left(r,c\right)=\tau +B\left(r,c\right)$ where *τ* is a user set gray value threshold. Given $F_{t}$ the contours $c_{t}^{i}$ of the animals are calculated by using the algorithm proposed in [14] resulting in a set of contours $C_{t}=\left\{c_{t}^{1},c_{t}^{2},...,c_{t}^{N_{t}}\right\}$. *N*_*t*_ might differ from *N* since animals can be in contact with each other (leading to merged contours) or impurities on the substrate which are not included in the background image lead to artifacts. However, the contours in *C*_*t*_ can be filtered to identify single animals by assuming that all imaged animals cover approximately the same area. The filtered set ${\tilde{C}}_{t}$ of contours is given by

$${\tilde{C}}_{t}=\left\{c_{t}^{i}\;|\;\lambda_{\text{min}}\leq A\left(c_{t}^{i}\right)\leq \lambda_{\text{max}}\text{,}\;i=1,\ldots ,N_{t}\right\}$$

where *λ*_min_ < *λ*_max_ are two user defined thresholds and $A(c_{t}^{i})$ is the contour area given by the number of pixels enclosed by $c_{t}^{i}$. Both the contours that fulfill $A\left(c_{t}^{i}\right)>\lambda_{\text{max}}$ which are assumed to represent colliding animals and contours with $A\left(c_{t}^{i}\right)<\lambda_{\text{min}}$ which are assumed to be artifacts are ignored in further calculations.

#### Model extraction

**Fig 2:**
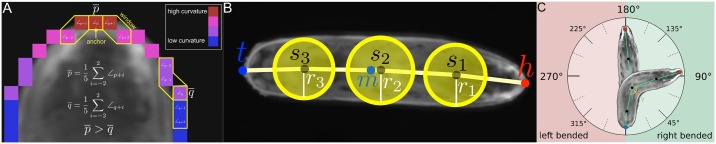
Calculation of the animal representation. (**A**) Example of the sliding window algorithm for points $\bar{p}$ and $\bar{q}$ with a window size of 5. Contour points with sharp angles are given in red. (**B**) Animal representation including the notation given in the text. (**C**) Body bending is calculated based on Eq (3). An animal is not bended if *γ* = 180°, bended to the left if *γ* > 180° and bended to the right if *γ* < 180°.

For each contour $c_{t}^{i}\in {\tilde{C}}_{t}$ we compute a model representation of the associated animal. First, the spine is calculated based on curvature values which are obtained for each point **p** on the contour using the first pass of the IPAN algorithm [15]. Given all curvatures ∠_**p**_ the two regions with sharpest acute angle are located using a sliding window approach as illustrated in Fig 2a. The point with the sharpest overall mean angle is identified as head **h** and the point with the second sharpest mean angle (with an appropriate distance *δ*_*h* ↔ *t*_ to **h**) is identified as tail **t**. This assignment is done during the model extraction for each frame individually. In order to avoid head and tail switches and to ensure a reliable identification of these points for different organisms like larvae or C. elegans, the positions of the head and tail are refined in a post processing step using posture and motion features over time (see Post processing section).

The initial **h** and **t** identification is used to split the contour into two halves $c_{t,1}^{i}$ and $c_{t,2}^{i}$. Without loss of generality, let $|c_{t,1}^{i}\left|<\right|c_{t,2}^{i}|$. Then $c_{t,2}^{i}$ is re-sampled, so that $|c_{t,1}^{i}\left|=\right|c_{t,2}^{i}|$ by utilizing linear interpolation between points in $c_{t,2}^{i}$. Now each point $p_{1}^{j}\in c_{t,1}^{i}$ corresponds to a unique point $p_{2}^{j}\in c_{t,2}^{i}$. The spine points **s**_1_, …,**s**_*L* − 2_ are calculated by determining *L* − 2 equidistant pairs $\left(p_{1}^{j},p_{2}^{j}\right)$ along $c_{t,1}^{i}$ and $c_{t,2}^{i}$ and by setting $s_{j}=\frac{p_{2}^{j}+p_{1}^{j}}{2}$. The radii *r*_*j*_ are calculated similarly by $r_{j}=\frac{∥p_{2}^{j}-p_{1}^{j}∥}{2}$. In addition, the center of mass **m** is calculated based on $c_{t}^{i}$. As a result, each animal *i* at time *t* is defined by a model

$$l_{t}^{i}=\left(h,\left(s_{1},r_{1}\right),\ldots ,\left(s_{L-2},r_{L-2}\right),t,m\right)$$ (1)

which is depicted in Fig 2b.

#### Tracking

Considering a sufficient spatio-temporal resolution tracking can be done by assigning animals between consecutive frames: all active animals $L_{t}=\left\{l_{t}^{i}|i=1,\ldots ,N_{t}\right\}$ at time *t* need to be associated with either one detected animal from the set $L_{t+1}=\left\{l_{t+1}^{j}|j=1,\ldots ,N_{t+1}\right\}$ at time *t* + 1 or removed from the set of active animals. Mathematically this assignment is known as a bipartite matching between $L_{t}$ and $L_{t+1}$. Let the costs for an assignment between an animal *i* at time *t* and an animal *j* at time *t* + 1 be *κ*_*ij*_. This leads to the following cost matrix:

$$C_{t}=\begin{array}{l} l_{t}^{1}\\ l_{t}^{2}\\ \vdots \\ l_{t}^{N_{t}} \end{array}\left(\begin{array}{llll} l_{t+1}^{1} & l_{t+1}^{2} & \cdots & l_{t+1}^{N_{t+1}}\\ \kappa_{11} & \kappa_{12} & \cdots & \kappa_{1N_{t+1}}\\ \kappa_{21} & \kappa_{22} & \;\cdots & \kappa_{2N_{t+1}}\\ \vdots & \vdots & \ddots & \vdots \\ \kappa_{N_{t}1} & \kappa_{N_{t}2} & \cdots & \kappa_{N_{t}N_{t+1}} \end{array}\right)\in {\mathbb{R}}^{N_{t}\times N_{t+1}}$$

**Fig 3:**
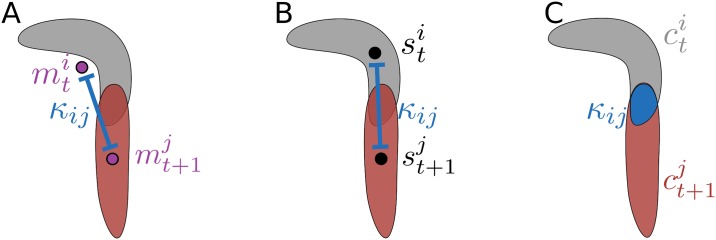
Different cost measurements. (**A**) Center of mass-based cost. (**B**) Spine-based cost. (**C**) Contour-based cost.

The following three cost measurements are implemented in FIMTrack:

1. Euclidean distance between the center of masses (Fig 3a): $\kappa_{ij}=\left|\right|m_{t}^{i}-m_{t+1}^{j}{\left|\right|}_{2}$
2. Euclidean distance between the mid-points (Fig 3b):$\kappa_{ij}=\left|\right|s_{\frac{L}{2},t}^{i}-s_{\frac{L}{2},t+1}^{j}{\left|\right|}_{2}$
3. The intersecting area in pixels given two consecutive contours (Fig 3c)

Since the center of mass is extracted from the contour, the translation between consecutive frames is very smooth. In contrast, the middle spine point contains more jitter, but is forced to be inside the contour. Costs derived from the overlap of two consecutive contours fulfill both criteria as they are smooth and based on pixels within the contours.

To solve the aforementioned assignment problem two algorithms are implemented in FIMTrack. One is the Hungarian algorithm [16, 17] which has one drawback: Given distance-based costs, the algorithm will find assignments for all $\tilde{n}=\min(N_{t},N_{t+1})$ animals. For example, if animal *i* disappears at time *t* while another animal *j* appears at time *t* + 1, the algorithm will assign these two animals even if the Euclidean distance between them is very large. Thus, we check each assignment if at least one point of the model of animal *i* is inside the contour of animal *j*. Otherwise animal *i* is considered to be inactive, the associated trajectory is terminated, and animal *j* is initialized as a new animal.

The second algorithm follows the greedy pattern. Given $L_{t}$ and $L_{t+1}$ the algorithm determines sequentially for each animal $l_{t}^{i}\in L_{t}$ the best matching animal $l_{t+1}^{j}\in L_{t+1}$ given one cost measurement (note that each match has to be unique). To exclude irrational assignments this algorithm requires an additional threshold *τ*_greedy_ specifying the maximal distance between two consecutive points if distance-based costs are used or the minimal amount of overlap in case of contour-based costs.

**Fig 4:**
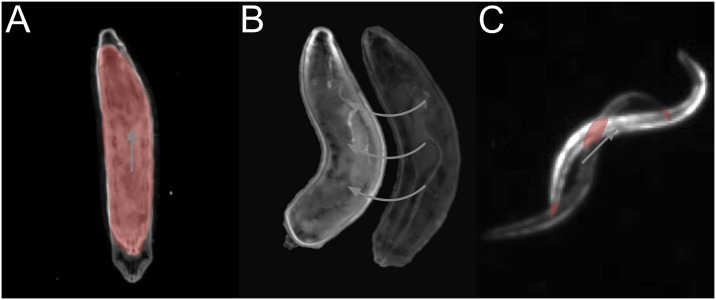
Examples of different assignment strategies. Overlapping regions are given in red and locomotion is indicated by arrows. (**A**) During forward locomotion of Drosophila larvae the association can be done using overlap-based costs and the Hungarian algorithm. (**B**) Given rolling behavior of Drosophila larvae, an assignment using contour overlaps as costs for the Hungarian algorithm might fail due to inappropriate frame rates. (**C**) For tracking the snake-like motion of C. elegans, contour-based assignments might be insufficient.

The possibility to choose between multiple costs and optimization algorithms extends the range of organisms which can be analyzed even at various spatial and temporal resolutions. For example, during peristaltic forward locomotion of Drosophila larvae, the Hungarian algorithm in combination with overlap-based costs is feasible to associate the larvae over time (Fig 4a). In contrast, rolling larvae with unsuitable temporal resolution lead to false assignments using the Hungarian algorithm with overlapping contour costs: due to strong changes in the body bending and the relatively fast lateral locomotion no overlaps can be detected within contours of consecutive frames (Fig 4b). Similarly, C. elegans moves in a snake like motion so that contour-overlap-based assignments may fail (Fig 4c).

After processing all frames, the overall path of an animal *i* is given by

$$L^{i}=\left(l_{t_{1}}^{i},l_{t_{1}+1}^{i},\ldots ,l_{t_{2}}^{i}\right)$$ (2)

where *t*_1_ specifies the first frame the animal appears at and *t*_2_ indicates the last valid measurement (1 ≤ *t*_1_ ≤ *t*_2_ ≤ *T*; *T* represents the total number of frames).

#### Post processing

The initial definition of the larval orientation is based on two regions with the sharpest acute angles. Due to the non-rigid body wall or low per-animal resolutions this assumption is not true in some frames leading to alternating head/tail assignments. Furthermore, other model organisms like C. elegans can imply other curvature characteristics compared to larvae which leads to the necessity to correct the initial **h** and **t** calculation.

To adjust the assignments in $L^{i}$ (Eq 2) first distinct sequences $\tilde{L^{i}}\subseteq L^{i}$ where the animal is not coiled are determined. Afterwards, a probability indicating whether the head/tail assignment is correct or not is calculated for all tuple $l_{t}^{i}\in \tilde{L^{i}}$ based on the following constrains:

1. Locomotion conformity (i.e. the mid-point/head vector points in the direction of the locomotion)
2. Bending conformity (i.e. larvae move the head but not the tail during reorientations)

If the head and tail are swapped, the spine points **s**_*i*_ and the radii **r**_*i*_ are reversed, too. Furthermore, all spine-point derived features are recalculated. Note that, although these constraints are derived from larval locomotion, the resultant probability is still valid if a C. elegans worm moves forward in more than 50% of the frames. It is worth mentioning that, after identifying **h** and **t**, the position of these points along the spine is fixed by assigning each subsequent head/tail point based on the respective predecessor with smallest Euclidean distance. Furthermore, even if the head and tail are swapped one click in the results viewer module is sufficient to correct the model throughout the entire recording (see Results reveiwer module).

#### Feature calculation

To quantify the locomotion in more detail several primary, secondary, and motion-related features are calculated by FIMTrack.

*Primary features*. In addition to the representation of an animal $l_{t}^{i}$ (Eq 1) the area *A* and the perimeter *P* are calculated.

*Secondary features*. The main body bending angle *γ* is calculated based on the head **h**, the mid spine point $s_{\frac{L}{2}}$, and the tail **t**. Given the vectors $v_{1}=h-s_{\frac{L}{2}}$ and $v_{2}=s_{\frac{L}{2}}-t$, Eq (3) is used to calculate the bending in degree.

$$\gamma =\arccos\left(\langle \frac{v_{1}}{∥v_{1}∥},\frac{v_{2}}{∥v_{1}∥}\rangle \right)\cdot \frac{180^{{}^{\circ}}}{\pi }$$ (3)

As a consequence, an animal is not bended if *γ* = 180°, bended to the left if *γ* > 180° and bended to the right if *γ* < 180° (Fig 2c). Since a user-specified number of spine points can be extracted these points can be used to extract other bendings along the spine by using Eq (3) with appropriate **v**_**1**_ and **v**_**2**_ (e.g. to quantify the stereotypical *S*-shape of C. elegans).

Given a threshold *τ*_bend_, an animal sweeps to the left if *γ* ≥ 180° + *τ*_bend_ and sweeps to the right if *γ* ≤ 180° − *τ*_bend_. Furthermore, the spine length *S*_*l*_ is calculated by summing up the Euclidean distances between the head, all spine points, and the tail:

$$S_{l}={∥h-s_{1}∥}_{2}+\sum_{i=1}^{L-3}{∥s_{i}-s_{i+1}∥}_{2}+{∥s_{L-2}-t∥}_{2}$$

**Fig 5:**
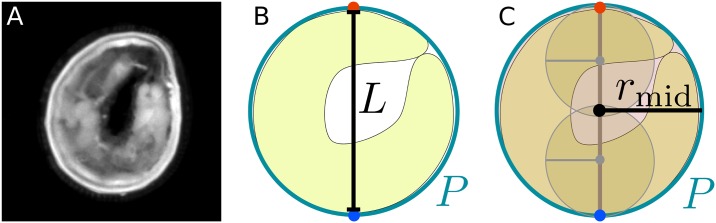
Two constraints are used to determine if an animal is in a coiled state. (**A**) Larva in a coiled state. (**B**) Perimeter *P* to spine length *L* fraction. (**C**) Mid spine point radius *r*_mid_ to perimeter *P* ratio.

In case of coiled animals (Fig 5a) the spine calculation fails to extract the posture correctly. As a consequence, all spine-related features are not reliable. To mark these ambiguous situations, a binary indicator *c*? is introduced to determine coiled states. The coiled indicator is true if one of the following constraints is satisfied:

1. The perimeter to spine length fraction converges to *π* ($\frac{P}{L}\approx \pi$; Fig 5b)
2. The circle given by the mid spine point radius divided by the perimeter converges to 1 ($\frac{2r_{\text{mid}}\pi }{P}\approx 1$; Fig 5c)

*Motion-related features*. Most of the motion-related features are calculated based on the animal’s center of mass **m** since this point is calculated directly from the contour and does not depend on the spine calculation. The accumulated distance for an animal at time *t* is calculated by

$$d_{\text{acc}}=\sum_{i=1}^{t-1}{∥m_{i}-m_{i+1}∥}_{2}$$

and the distance to origin is given by

$$d_{\text{org}}={∥m_{1}-m_{t}∥}_{2}$$

Furthermore, the velocity for time *t* is calculated by

$$v_{m}=\frac{∥m_{t-\frac{\text{fps}}{2}}-m_{t+\frac{\text{fps}}{2}}{∥}_{2}}{\text{fps}}$$

where fps is the given frame rate. In a similar fashion, the acceleration is obtained by using consecutive velocities:

$$a_{m}=\frac{∥v_{m,t}-v_{m,t+1}{∥}_{2}}{\text{2}}$$

To identify if an animal is in a go phase, a binary indicator *g*? is used. The following constraints must be valid for a go phase:

- The velocity *v*_*m*_ is above a certain threshold
- There is no strong bending *γ* of the animal’s body

To avoid alternating go phase measurements, a user-specified minimal go phase length (*τ*_go_) is used to extract continuous phases. This implies that the number of consecutive *g*? = *true* measurements has to be ≥ *τ*_go_ to classify this sequence as a go phase. An animal is in a reorientation phase if *g*? is false.

*Stimulus-related features*. In order to extend the capabilities of FIMTrack it is possible to place different stimulus markers on the raw image in the results viewer. The following markers are supported:

- **Point** which is a (*x*, *y*) position
- **Line** which is a straight line segment
- **Rectangle** which is an arbitrarily sized axis-aligned 2D rectangle
- **Ellipsoid** which is an arbitrarily sized axis-aligned 2D ellipsoid

For all markers the additional features *distance to stimulus*, *bearing angle to stimulus*, and *is in stimulus region* are calculated for each time point and animal. The distance to a stimulus is given by the Euclidean distance between the center of mass **m** of the animal and the point **p** representing the nearest point on the stimulus. The bearing angle *β* is obtained by using Eq (3) with **v**_**1**_ = **m** − **t** and **v**_**2**_ = **p** − **t** (note that the tail **t** is used since it is not affected by head casts).

Given a **point stimulus** the necessary computations are straight forward. The nearest point between the animal and a **line stimulus** is obtained by performing an orthogonal projection of **m** on the line defined by the line segment. If the projected point **p** is not located on the line segment we take one of the two endpoints of the line segment as the nearest point which has the minimal Euclidean distance to **p**. In case of a **rectangular stimulus** the nearest point **p** is calculated by **p** = arg min_**p**_*i*__ ∥**m** − **p**_*i*_ ∥_2_, where **p**_*i*_ is the associated orthogonal protection of **m** onto each of the four boundaries of the stimulus. Since the calculation of the exact nearest point **p** of **m** on a **ellipsoid stimulus** cannot easily be performed in an analytical fashion, we use an approximation of **p**. Given an axis-aligned ellipse centered at **c** = (*c*_*x*_, *c*_*y*_). First, a line **l** going through **m** and **c** is determined. Next the intersection points **p**_**1**_ and **p**_**2**_ between **l** and the ellipse are obtained and the intersection point with the minimum distance to **m** is taken as the approximation of **p**.

### Results viewer module

**Fig 6:**
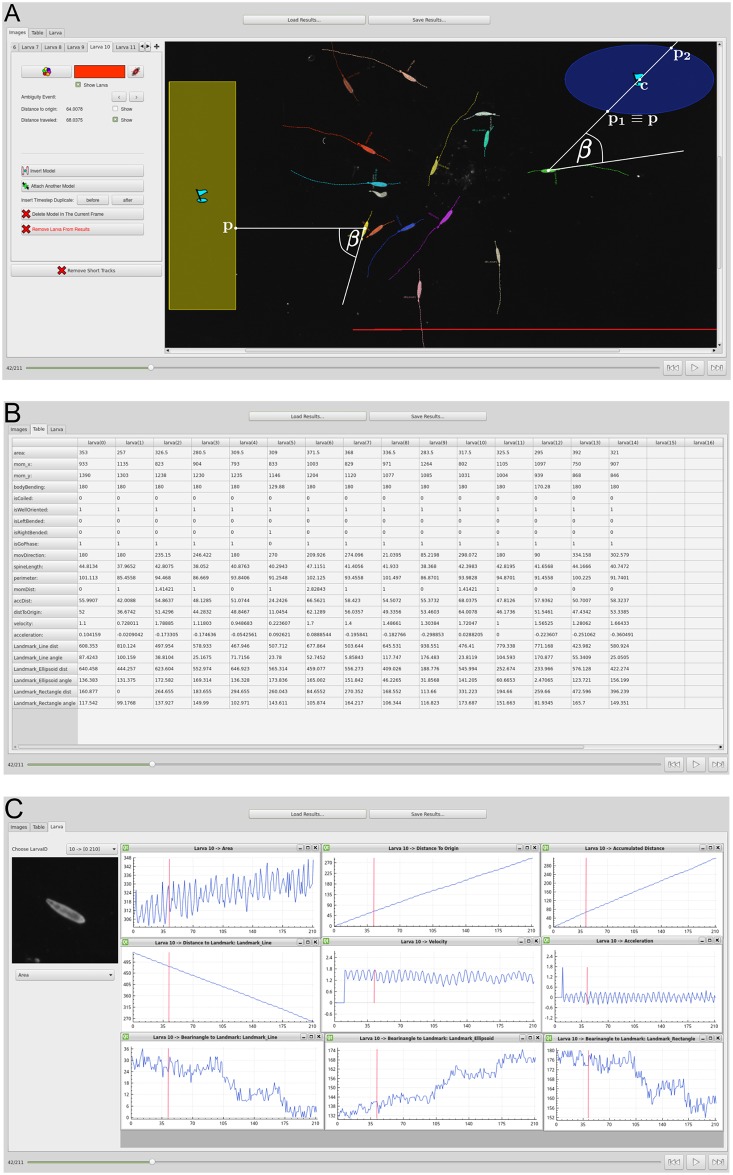
Results viewer module. (**A**) Image view with the raw image, an overlay of the color coded features, and two stimuli marker with the notations given in the text. (**B**) Table view. (**C**) Animal view with both a cropped region of a single larva and plots of some features.

The *results viewer* module offers the possibility to review the calculated features. The experimenter can load, display, and manually correct the posture and motion-related features or even manually track some animals if they could not be recognized automatically. If an animal model is adjusted manually all features are updated accordingly. The results viewer module itself is divided into three main parts, namely the *image view* (Fig 6a), the *table view* (Fig 6b) and the *animal view* (Fig 6c).

The image view provides a qualitative impression of the tracking results. Most of the calculated features are plotted color coded for each animal as an overlay onto the raw images. Moreover, the user can manually change the calculated model or merge/remove trajectories. This is particularly helpful to resolve ambiguous situations like coiled or colliding animals. In the table view all calculated features can be inspected in a table showing animals in columns and the associated features in rows. The animal view can be used to inspect the results for a single animal in more detail: both a cropped region of a single animal and plots for relevant features can be obtained simultaneously.

### IO module

This module is responsible for reading and writing files. Currently the image file formats TIFF and PNG are supported. A CSV file containing all calculated features, a YML file including the same measurements as the CSV file in combination with some additional informations like the processed images, and an image with color coded trajectories are generated after tracking has been performed. It should be mentioned that the CSV format is standardized and can directly be imported into a variety of analysis programs (e.g. MATLAB, Excel, R).

## Results

Most of the calculated features rely on a precise calculation of the center of mass (e.g. accumulated distance, velocity, bearing, etc.). Furthermore, good candidates to assess the quality of the calculated model are the mid spine point and the body bending (most of the secondary and motion-related features are derived from the underlying model). Here, we evaluate the accuracy of the software regarding these features.

### Ground truth data

**Fig 7:**
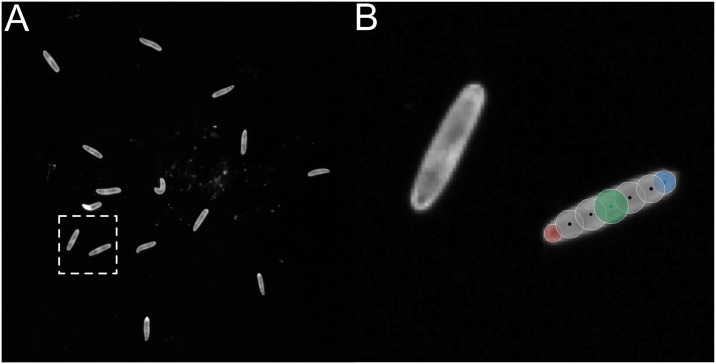
An image from the ground truth dataset and the manually generated model. (**A**) Exemplary image used for ground truth generation. (**B**) Close up of the dashed box from Fig 7a.

An image sequence with a resolution of 2040 × 2048 pixels acquired with a Basler acA2040-25gm camera equipped with a 16mm objective (KOWA LM16HC) containing 15 larvae over 211 frames was used to generate ground truth data (Fig 7a and 7b). All animals were associated with a larval model consisting of the head, tail, and 5 equidistant spine points associated with appropriate radii. Furthermore, the center of mass was calculated based on these models.

For the subsequent analysis we considered the 10 trajectories of the larvae which could be tracked by FIMTrack over all 211 frames (i.e. larvae which crawl at least by there own body length and do not collide).

### Measured deviations

Deviations from the ground truth are determined by calculating the Euclidean distances between the tracking results and ground truth data for both the center of mass and the central spine point. For the body bending, absolute differences are used to determine the accuracy. Table 1 illustrates the deviations.

**Table 1:** Deviations for the examined parameters. Body bending is given in degree, all other parameters are given in pixels. Max^⋆^ represents the values obtained by including outliers whereas OL gives the number of outliers.

Deviations	Mean (±Std)	Median	Min	Max	Max^⋆^	OL
Center of mass	1.86 (±0.23)	1.85	1.21	2.55	2.84	17 (0.80%)
Central spine point	1.84 (±1.10)	1.57	0.50	4.58	16.84	61 (2.89%)
Body bending	3.54 (±7.51)	2.55	0.00	13.16	171.00	46 (2.18%)

Obviously the deviation of the center of mass and the deviation of the central spine point are below 2 pixels in mean and median. It should be noted that during tracking no sub-pixel accuracy is used and thus the minimum possible error is 1 pixel if the displacement happens either in *x* or *y* direction. For a diagonal displacement the minimum possible error is $\sqrt{2}\approx 1.41$ pixels. In combination with the area of the animals which range from 232.50 to 454.50 square pixels, this leads us to the assertion that deviations below 2 pixels are caused due to noise.

#### Center of mass

**Fig 8:**
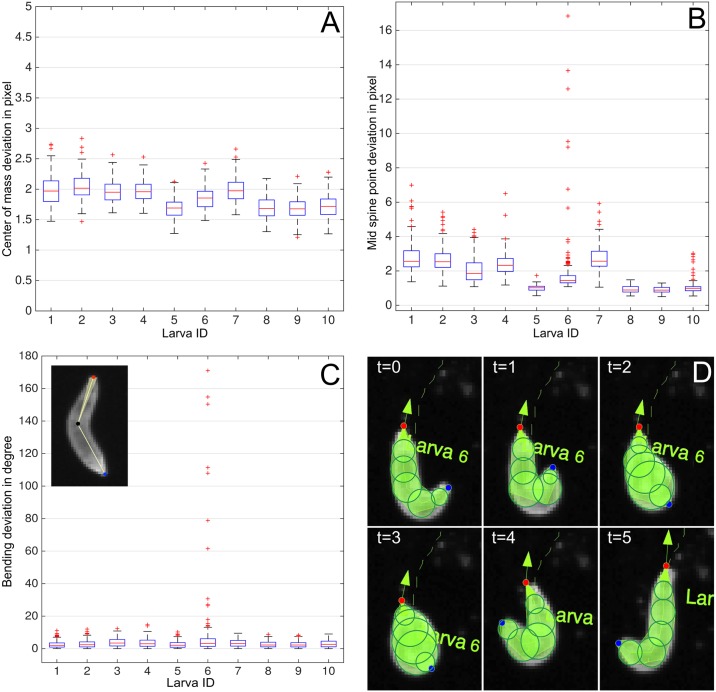
Measured deviations. (**A**) Center of mass deviations. (**B**) Central spine point deviations. (**C**) Body bending angle deviations. The mean divergence of the body bending is sketched by the light yellow area in the larva image at the top left corner. (**D**) The coiled structure of larva 6 (at *t* = 2, 3) causes outliers in the measurements (compare to Table 1). The head is given in red and tail is given in blue.

A detailed overview of the center of mass progress is given in Fig 8a. Each boxplot represents the center of mass deviation for the respective larva. None of the measurements has a median deviation above 3 pixels. This suggests that the divergence is rather the result of an inaccuracy of the tracking algorithm but more likely caused by the previously performed image processing and definitely influenced by the non-contour-based center of mass extraction in the ground truth data.

#### Central spine point

The central spine point location contains more outliers compared to the center of mass measurements. The maximal deviation (including outliers) between a measurement and the ground truth is 16.84 pixels (Table 1). As illustrated in Fig 8b measurements for larva 6 include most outliers. These inaccuracies are caused within several frames in which the animal is coiled resulting in an erroneous spine calculation (Fig 8d). The median spine point deviation is below 2 pixels and after removing the outliers the maximum distance decreases to 4.58 pixels (Table 1).

**Fig 9:**
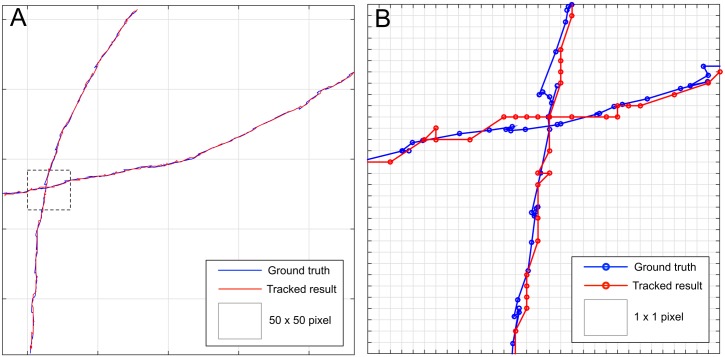
Resultant center of mass trajectories compared to the ground truth paths. (**A**) Center of mass point of the ground truth and tracked larvae. (**B**) Close-up or the dashed box from Fig 9a.

To further study the accuracy an overlay of ground truth and calculated central spine point trajectories is given in Fig 9. Since no sub-pixel accuracy is used for tracking, the calculated path contains more straight lines interrupted by edges. However, the deviation from the ground truth path is rarely more than one pixel.

#### Body bending

A high tracking precision can also be observed within the body bending quantification: the mean deviation is below 4° (Table 1) which is depicted in the top left corner of Fig 8c where the head of the larva is given in red, the tail in blue, and the central spine point in black. The mean deviation is indicated by the light yellow area visible at both sides of the spine segment connecting the head and the tail.

By taking a closer look at the plots given in Fig 8c, it can be seen that again only larva 6 includes several frames with a very strong deviation. Since the deviations go up to 171° the head and the central spine point are swapped which can be observed in Fig 8d.

## Availability and future directions

FIMTrack is freely available as a pre-built binary package for Windows and Mac at http://fim.uni-muenster.de. Further documentation and exemplary FIM-images for testing purposes are available at the same website. An open-access video tutorial for experimental biologists illustrating the usage of our system with and without stimulation can be found in [12].

The source code of FIMTrack is licensed under the GNU GPLv3 and can be obtained from https://github.com/i-git/FIMTrack. Users implementing new features or extensions are encouraged to submit their work via GitHub’s pull request mechanism for inclusion in a coming release.

In the past, several others groups successfully used FIMTrack to differentiate between different behavioral phenotypes (examples for Drosophila larvae can be found in [9, 18, 19] and for C. elegans in [13]). Furthermore, the software has been used as the basis for extensions in order to address more specific biological questions [8, 10, 20].

It should be noted that FIMTrack has been initially developed for FIM images and Drosophila larvae [7]. For example, the algorithms described above only segment the animals if the background is darker than the foreground (i.e. the animals). However, we successfully adopted the algorithm to track images recorded with transmitted light illumination by inverting the images before passing them to FIMTrack. Furthermore, some of the extracted features are only valid for larval behavior like the stop and go classification. Otherwise, since the complete model of the animal (Eq 1) obtained after tracking, prepossessing, and maybe some user adjustments is saved in a standardized file format (i.e. CSV), higher-level features for other model organisms can be derived easily. Finally, FIMTrack does not include a module to resolve colliding animals so that the identities of animals participating in a collision get lost and the trajectories of these animals terminate. After the ending of the collision the associated animals receive new identities and are treated as newly appeared.

In the future, we are going to extend FIMTrack by optimizing the tracking for other model organisms like flatworms. In order to overcome the problem of losing identities and behavioral quantities during animal-animal contact, we are working on a statistical approach capable of resolving colliding animals.

## Supporting information

S1 Data In order to quantify the accuracy of FIMTrack we have manually tracked 15 larvae over 211 frames. The resultant quantities and the used evaluation script are provided in order to guarantee reproducibility of our results. Note that the images can be downloaded at http://fim.uni-muenster.de.
(ZIP)

S1 Text FIMTrack manual describing the work flow. (PDF)
