# Supplementary material for: FIMTrack: An open source tracking and locomotion analysis software for small animals
Source: PLoS Comput Biol. 2017 May 11;13(5):e1005530. doi: 10.1371/journal.pcbi.1005530 (PMC5444858; doi:10.1371/journal.pcbi.1005530)
Supplement: S1 Text — (PDF) [file pcbi.1005530.s002.pdf]

FIMTrack Manual

Contents

1 Installation Instructions 3

2 How To Use FIMTrack 3

2.1 How To Track . . . 3
2.2 How To Adjust The Preferences . . . 5
2.3 Set Region Of Interest . . . 8
2.4 Start Tracking . . . 8
2.5 Batch Processing . . . 9
2.6 Output Of FIMTrack . . . 9
2.7 View Results using FIMTrack . . . 9
2.8 Manual Tracking . . . 10
2.9 Insert Stimuli . . . 14
2.10 Save Adjusted Results And Stimuli . . . 14

3 Features Calculated By FIMTrack 14

3.1 Primary Features . . . 14
3.2 Secondary Features . . . 16
3.3 Tertiary Features . . . 16

4 Contact 16

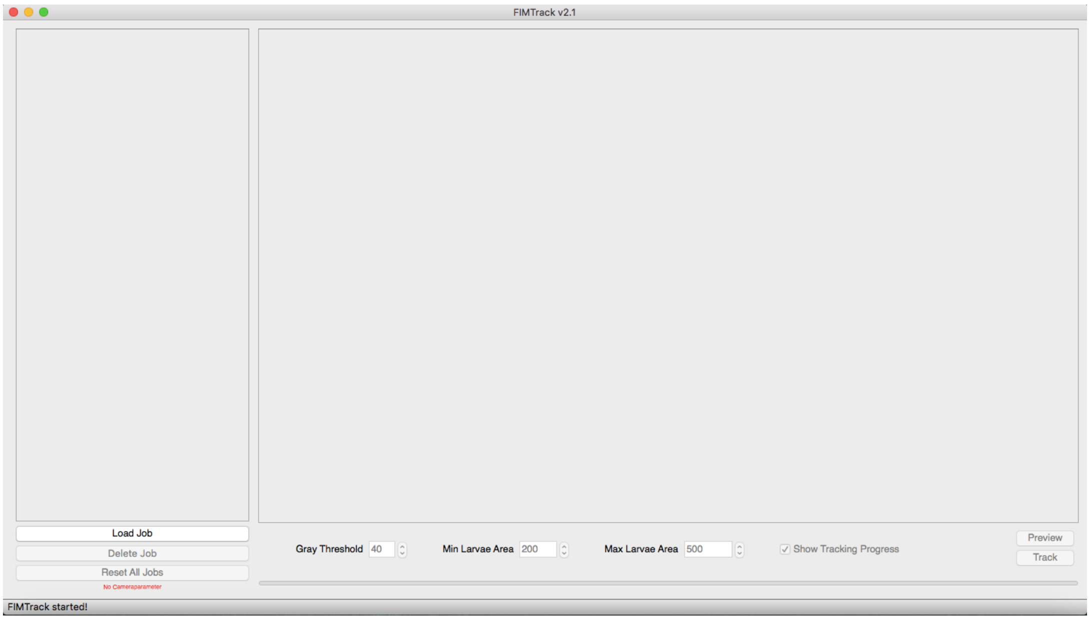

Figure 1: Main graphical used interface of FIMTrack.

Introduction

FIMTrack is a tracking program primarily designed for acquiring locomotion trajectories of *Drosophila melanogaster* larvae but it is also possible to analyze the behavior of other small model organism such as C. elegans. The software is optimized for FIM images. FIM is an acronym for FTIR-based Imaging Method, whereby FTIR is the short form for Frustrated Total Internal Reflection. Details regarding the imaging technique can be found in [1, 2]. Furthermore, a video tutorial demonstrating the biological usability of the FIM system and FIMTrack can been found in [3].

The main focus in this document is on the *how-to* use the software and on providing an overview of all calculated features and parameters.

1 Installation Instructions

FIMTrack is available for the operating systems Windows, Mac, and Linux. The source code is available under the GNU GPLv3 at `https://github.com/i-git/FIMTrack` and pre-compiled binaries for Windows and Mac are available at `http://fim.uni-muenster.de`.

2 How To Use FIMTrack

2.1 How To Track

After starting FIMTrack the main graphical user interface (GUI) appears (Figure 1). To track larvae which were imaged using FIM imaging, first click the *Load Job* button, navigate to the folder containing the TIFF or PNG image files, and select the image files which you would like to analyze (Figure 2). **IMPORTANT: FIMTrack can only handle TIFF or PNG images.** If the images are not available in the required format, conversion can be done using Fiji[1], ffmpeg[2], or other third party software.

You can get a preview of an image by clicking on the image name inside the loaded jobs view on the left side of the GUI (Figure 3). Furthermore, you can zoom in and out by using *cmd/Ctrl + mouse wheel* or for faster zooming *cmd/Ctrl + Shift + mouse wheel* while our mouse courser is located over the preview image. If zooming is not possible, please click with the left mouse button in the preview image.

[1] `https://fiji.sc/`
[2] `https://ffmpeg.org/`

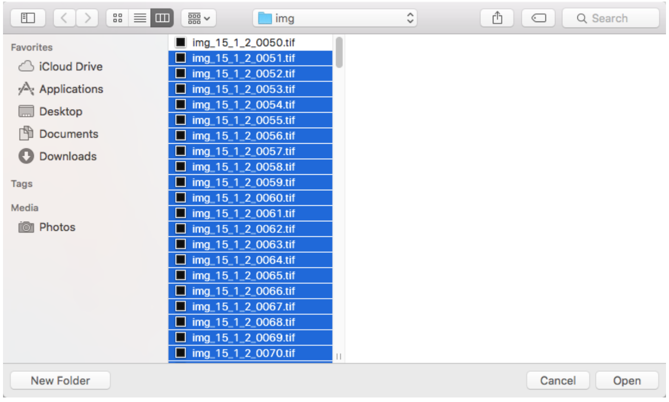

Figure 2: Select the images which should be load into FIMTrack.

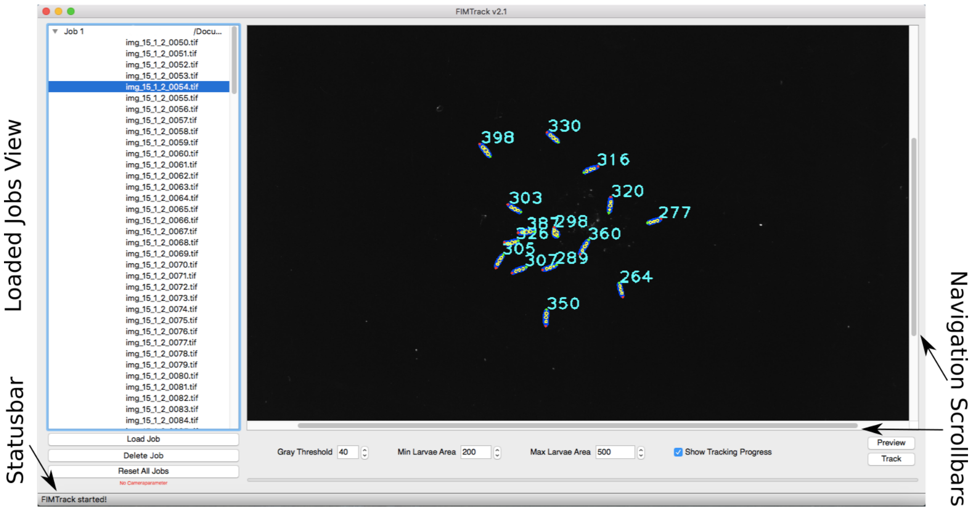

Figure 3: Preview image.

Navigation inside the preview image can be done by using the scrollbars around the image. The preview itself is useful to adjust the following parameters (at the bottom of the main GUI):

- **Gray Threshold** specifies the minimal brightness of the foreground objects (i.e., larvae). If no larval foreground object is highlighted in the preview image, the **Gray Threshold** parameter has to be decreased. If artifact which are darker compared to the foreground object are highlighted this parameter has to be increase.

- **Min Larvae Area** specifies the minimal amount of pixels covered by the larval contour. All objects which consist of less pixels are neglected in further process.

- **Max Larvae Area** specifies the maximal amount of pixels covered by the larval contour. All objects which consist of more pixels are neglected in further process.

A foreground object is considered to represent a larva if the associated contour is highlighted in blue in the preview image. Red, yellow, and green circles serve as additional markers in order to indicate a correct detection and segmentation of an animal. The head is marked in red, the tail is marked in green, and the thickness of a larva is approximated by yellow circles. The blue number next to a larva indicates the covered area in pixels (Figure 4a) in order to provide an initial estimation for the **Min** and **Max Larvae Area** parameters. **Since currently FIMTrack is not able to resolve colliding animals, it is important that the Max Larvae Area parameter is smaller than the area covered by two colliding animals.**

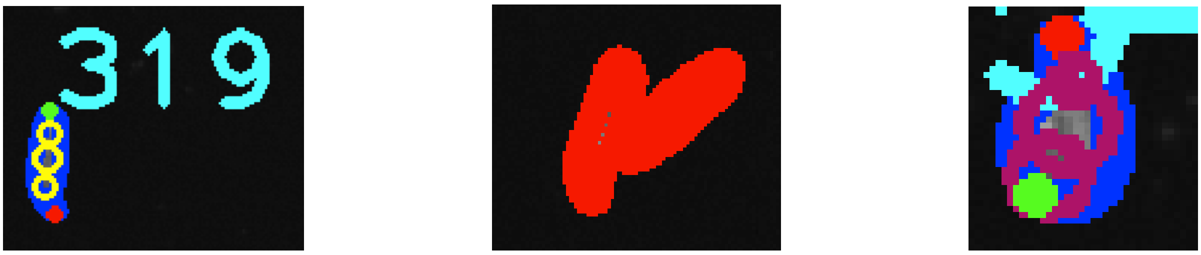

(a) (b) (c)

Figure 4: FIMTrack preview cropping. (a) Correct detected animal and its covered area. (b) Two colliding larvae exceeding the **Max Larvae Area** threshold. (c) Coiled larva (i.e., head is touching the tail).

If the area covered by larvae is smaller than the **Min Larvae Area** parameter value, reduce this parameter and select an image for preview from the loaded jobs view on the left side of the GUI or click on the *Preview* button at the bottom right of the GUI in order to refresh the preview. Detected objects highlighted in red occupies a larger area as allowed by the **Max Larvae Area** parameter value. (Figure 4b). Furthermore, coiled larvae (i.e., the head is touching the tail) are highlighted in the preview too. These animals are marked by purple circles inside the contour (Figure 4c).

2.2 How To Adjust The Preferences

Preferences can be adjusted by selecting *Edit* → *Preferences* from the menu bar. There are two types of preference panels:

- **Standard** preferences includes camera related parameters (i.e., the frames per second (fps) used for tracking) (Figure 5a).
- **Advanced** preferences include all tracking and feature extraction related parameters (Figure 5b).

Standard Preferences Before starting the tracking, adjust the frames per second rate which is given by the image acquisition rate of your camera. This parameter will be used later on for calculating parameters such as velocity or movement direction.

Advanced Preferences The advanced preferences are structured in groups. Each group can be edit by un-checking the *Use Default* or *Use Dynamic ... Calculation* check box.

- **Background Subtraction Parameters** to calculate a static background image where no animals are visible but which includes almost all immovable artifact present on the tracking surface.
  - **From Image** specifies the first image from the image sequence which should be used for calculating the background image.
  - **Offset** specifies the step size which should be used for background image calculation. A step size of two means that only every second image will be used.
  - **To Image** specifies the last image from the image sequence which should be used for calculating the background image.
- **Larvae Extraction Parameters** for larval feature calculation.
  - **Number of Spine Points** specifies the number of points equidistantly placed along the spine. This value has to be odd in order to guarantee a real mid-spine point.
  - **Coiled Recognition Parameters** to detect coiled structures.

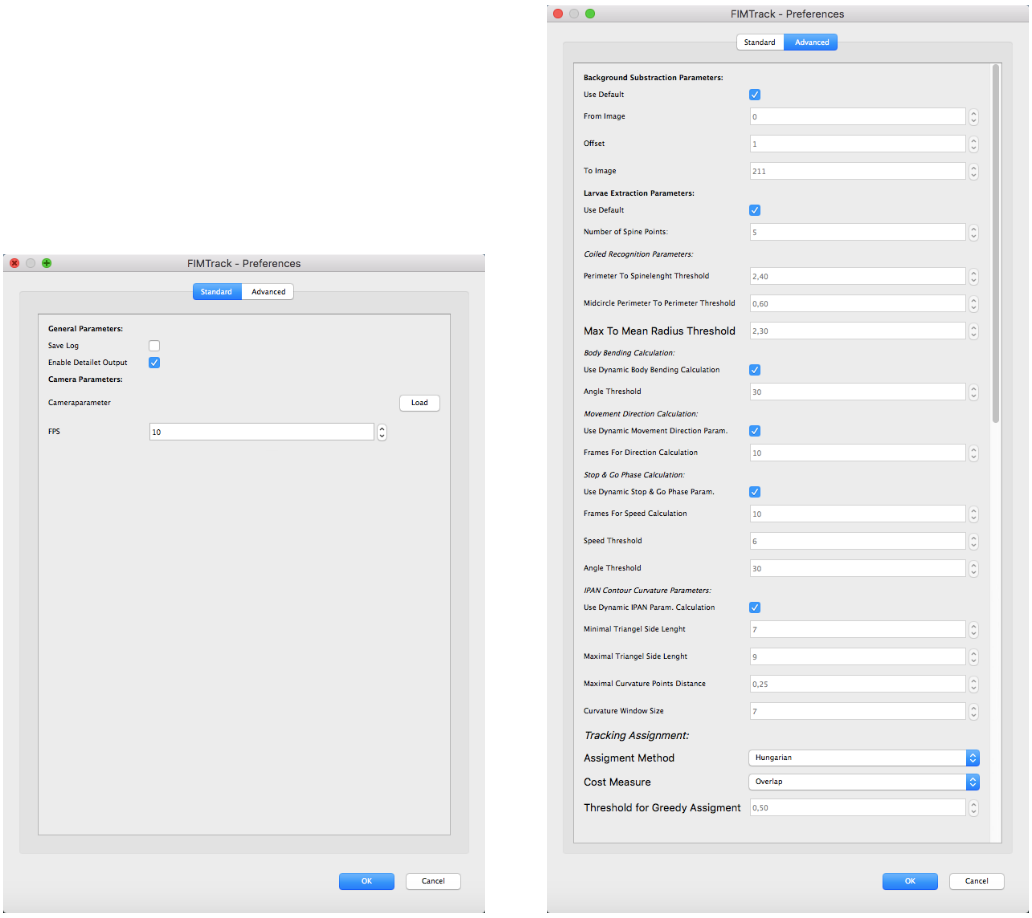

(a) (b)

Figure 5: FIMTrack preference panel. (a) Standard preferences dialog. (b) Advanced preferences dialog.

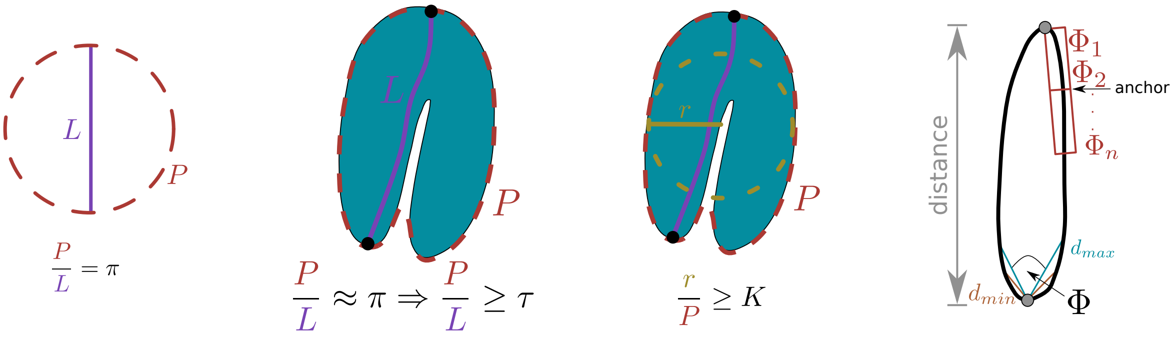

(a) (b) (c) (d)

Figure 6: Important parameters. (a) Ratio of a circle. (b) Ratio perimeter to spine length of a coiled larva. (c) Ratio mid-point radius to perimeter. (d) Parameters of the IPAN algorithm for calculating the spine (see text).

* **Perimeter To Spinelength Threshold** is used to identify coiled structures. A larva is in a coiled state, if the head is touching the tail. Given such situation the program cannot estimate distinct head and tail points (Figure 6b). Furthermore, in such cases, the ratio between the perimeter (P in Figure 6b) and the spine length (L in Figure 6b) converges to π (Figure 6a). Thus this ratio has to be above this threshold ($\frac{P}{L} \geq \tau$).

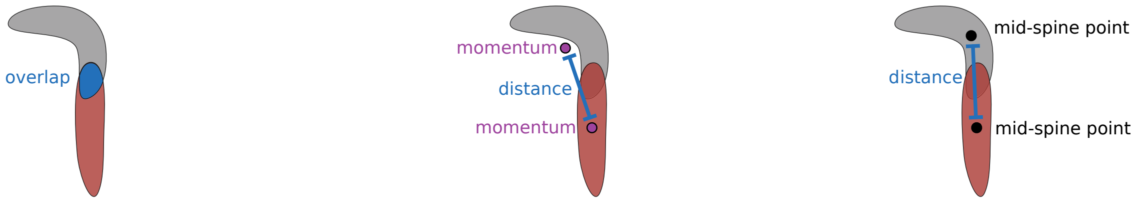

(a) (b) (c)

Figure 7: Different cost measurements. (a) Overlap based assignment cost measurement. (b) Assignment cost measurement based on the distance between the moments. (c) Assignment cost measurement based on the distance between the mid-spine points.

- * **Midcircle Perimeter To Perimeter Threshold** is used in a similar fashion like the **Perimeter To Spinelength Threshold**. If the ratio of the radius measured at the mid-spine point (r in Figure 6c) and the overall perimeter (P in Figure 6c) is above a given threshold K the animal is assumed to be in a coiled state (Figure 6c).

– **Body Bending Calculation** for head sweep detection.

  * **Angle Threshold** specifies the aberration of the main body bending (in both directions) to be detected as a head-sweep / bending (in degree).

– **Movement Direction Calculation**

  * **Frames For Direction Calculation**: given high frame rates (e.g. 10 frames per second), the movement detection cannot be calculated between consecutive frames since the traveled distance between consecutive frames is to short. Thus, the direction is calculated for every frame by looking at the larva in the current frame i and at the position of the same animal in the frame at $i+offset$, whereas this parameters specifies *offset*.

– **Stop & Go Phase Calculation** to classify stop and go phases.

  * **Frames For Speed Calculation**: given high frame rates (e.g. 10 frames per second), the correct speed cannot be calculated between consecutive frames since the traveled distance between two consecutive frames is to short. Thus, an offset is used to calculate the speed of an animal between frame i and $i+offset$, whereas this parameter specifies the *offset*.
  * **Speed Threshold**: given the **Frames For Speed Calculation** offset from above, this parameter specifies the minimal velocity a larva has to have during a go phase. Otherwise the larva is considered to be in a stop phase.
  * **Angle Threshold**: assuming that strongly bended animals cannot be in a go phase, this threshold specifies the maximal body bending angle which is acceptable during a go phase.

– **IPAN Contour Curvature Parameters** for calculating the spine of the animal. The IPAN algorithm proposed in [4] is used to calculate the curvature along the contour (Φ in Figure 6d). Therefore, a triangle is generate at each contour point.

  * **Minimal Triangle Side Length** specifies the minimal length of a triangle edge (in pixels; d_{min} in Figure 6d).
  * **Maximal Triangle Side Length** specifies the maximal length of the triangle edge (in pixels; d_{max} in Figure 6d).
  * **Maximal Curvature Points Distance** specifies the minimal distance the head and the tail point have to be apart (in pixels; distance in Figure 6d).
  * **Curvature Window Size** is used to smooth the calculated curvatures. The window size n is used to calculate the mean curvature at the anchor point (Figure 6d).

– **Tracking Assignment**: is used to extends the range of organisms which can be analyzed even at various spatial and temporal resolutions. For standard tracking, the Hungarian algorithm [5, 6] in combination with overlap-based costs should be used to associate the animals

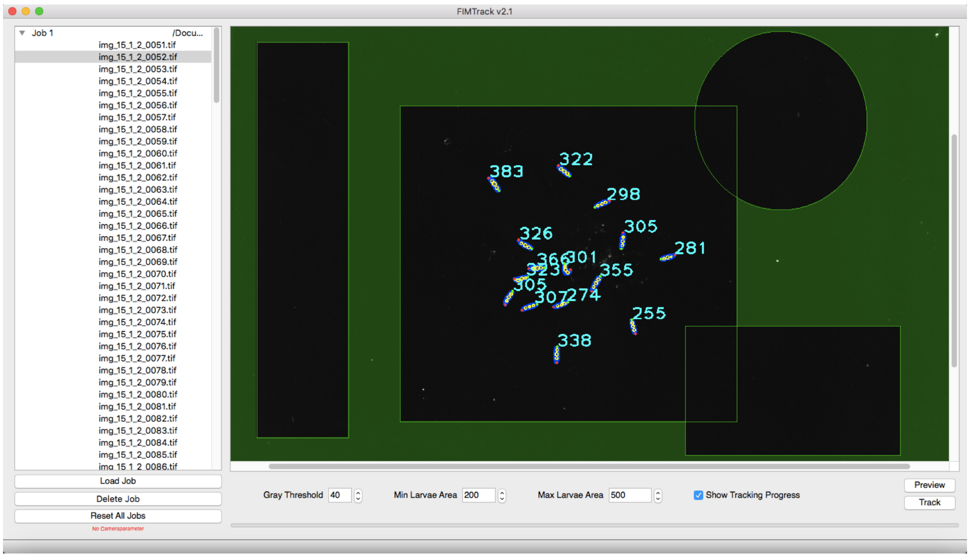

Figure 8: Several regions of interest are set.

over time. In contrast, an unsuitable temporal resolution or a relatively fast lateral locomotion of the animals lead to false assignments using the Hungarian algorithm with overlapping contour costs since no overlaps can be detected within contours of consecutive frames. Similarly, C. elegans moves in a snake like motion so that contour-overlap based assignments may fail.

- ∗ **Assignment Method**: you can choose between the Hungarian algorithm and an algorithm which follows the greedy pattern.
- ∗ **Cost Measure**: the following three cost measurements are available:
  1. The intersecting area in pixels given two consecutive contours (Figure 7a).
  2. Euclidean distance between the center of masses (momentum in Figure 7b).
  3. Euclidean distance between the mid-spine points (Figure 7c).
- ∗ **Threshold for Greedy Assignment**: specifying the maximal distance between two consecutive points if distance based costs are used or the minimal amount of overlap in case of contour-based costs. This parameter is only available if the greedy assignment method should be used.

2.3 Set Region Of Interest

You can mark an arbitrary number of image parts which should be included into tracking by defining so-called *region of interests* (Figure 8). Everything outside these regions will be neglected.

A region of interest is set by right clicking in the preview window. Select the shape of your preferred region (rectangular or ellipsoid) and assign a unique name for the region. You can move a region around by left clicking into the region and moving the mouse while holding the left mouse button down. Furthermore, the region can be resized by selecting the lower right corner of the region and by clicking and holding the left mouse button while moving the mouse (use *cmd*/*Ctrl* to highlight the corner). You can change the color of the regions by double-clicking at the colored *outside* of a region.

2.4 Start Tracking

If all relevant parameters are set up, click on the *Track* button in the main GUI (Figure 1) to start tracking. You will get intermediate results of the tracking process if the *Show Tracking Progress* check box is selected. **It is normal that after clicking *Track* nothing happens for some time. This is due to the background image calculation, which is performed prior to the tracking (see message in the statusbar at the lower left part of the main GUI).** If tracking is done, a short message will appear in the statusbar at the lower left part of the main GUI.

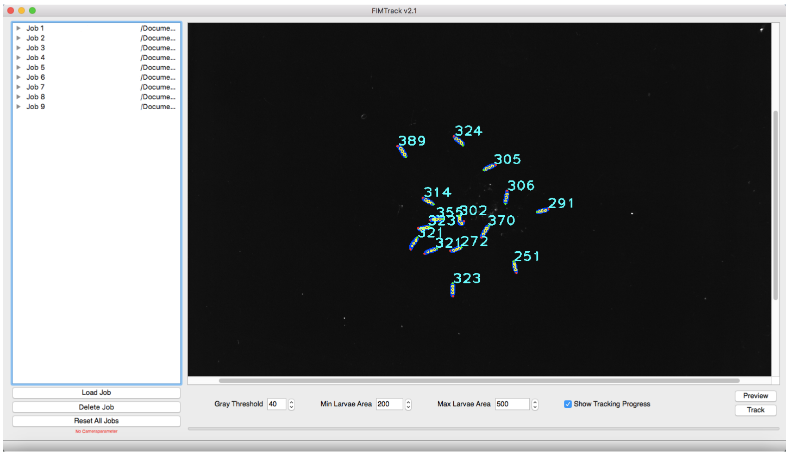

Figure 9: Several jobs are loaded for batch processing.

2.5 Batch Processing

If image capturing was done using the same settings (e.g camera and/or illumination settings), the resultant image sequences can be analyzed in a batch processing manner. Just add as many jobs as necessary using the *Load Job* button (Figure 9). Single jobs can be removed from the list using the *Delete Job* button. *Reset All Jobs* removes all jobs from the list.

2.6 Output Of FIMTrack

After tracking is done, FIMTrack creates a folder in the respective image folder and stores several files. The naming of the created folder is *output_date_time* where *date* is the current date and *time* is the current time. FIMTrack outputs:

- **table.cvs**: comma-separated values file containing all calculated features.
- **output.yml**: YAML files containing same features as the **table.cvs** file and some additional informations which will be used in the FIMTrack Results viewer (see below).
- **tracks.tif**: an image showing all computed trajectories which are color-coded. The spine is plotted for all animals and for all time points thus head sweeping can bee seen in these images.
- **tracksNoNumbers.tif**: similar to **tracks.tif** but without larvae identification numbers (IDs).

2.7 View Results using FIMTrack

FIMTrack offers the possibility to review the tracking results and thus the calculated features. Furthermore, it is also possible to correct the tracking results or to manually track several animals if they could not be tracked by the software. Select *Edit* → *Results viewer...* to open the *Results Viewer* (Figure 10).
Click on *Load Results...* to select both, images and tracking results. Please make sure to select **the same images** which were used for tracking (Figure 11a). After selecting the right images you will be ask to select the calculated tracking results, i.e., the *output.yml* file (Figure 11b). **It is important that you have selected the same images which were used for tracking. You can see the absolute image path and the image names which were used for tracking by opening the output.yml file with a text editor and check the first entries in this file.**

Using the Results Viewer The *Results Viewer* module has an *Image*, a *Table*, and a *Larva* tab to view the images and calculated features. Most of the calculated features are plotted color-coded onto the raw images at the image tab (Figure 12a). You can zoom in and out by using *cmd/Ctrl+mouse-wheel* or *cmd/Ctrl+shift+mouse-wheel*. By using the table tab you can inspect all calculated features of the larvae which were tracked at a specific time point (Figure 12b). The animal view can be used to inspect

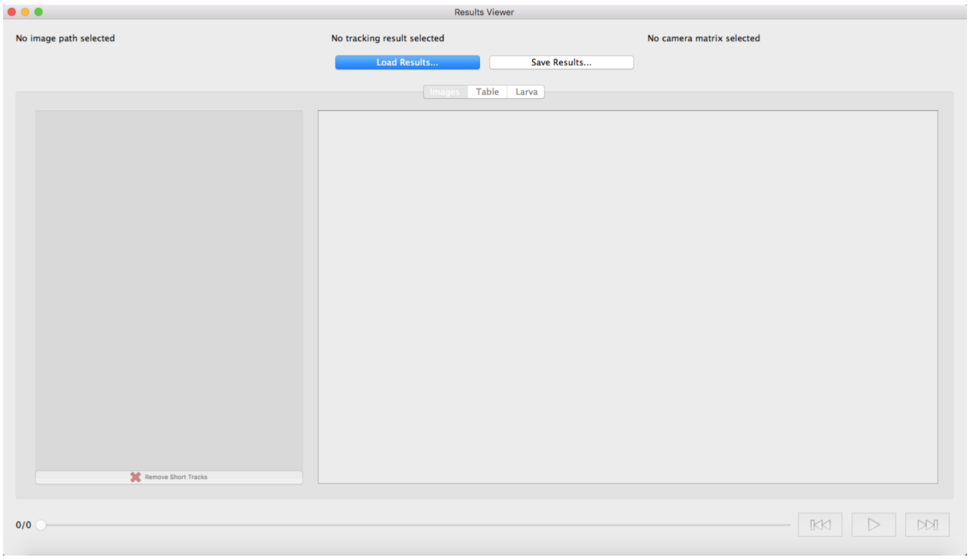

Figure 10: The Results Viewer of FIMTrack.

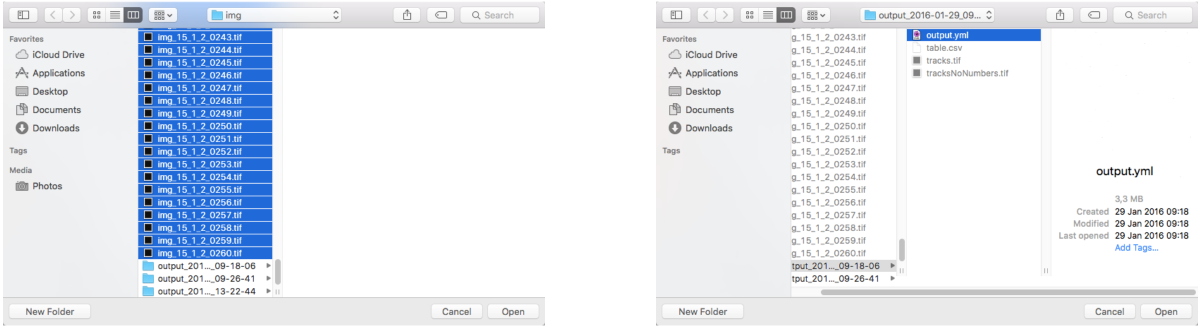

(a) (b)

Figure 11: Loading the results into the Result Viewer. (a) Select the same images which were used for tracking. (b) Select the calculated *output.yml* file.

the results for a single animal in more detail. A cropped region of a single animal is provided at the upper left part in this view. Furthermore, you have the opportunity to select some calculate features which should be plot from a dropdown menu in order to provide a first detailed view of the behavior of a single animal (Figure 12c).

Push the *play* button or use the slider at the bottom of the *Results Viewer* GUI to step through time. All currently available animal trajectories are drawn in the image tab. You can also use the left and right button to step from frame to frame.

2.8 Manual Tracking

At the larva tap you have the possibility to adjust the tracking results.

The Manual Tracking Panel The left panel in the image view offers the following options (Figure 13a):

1. Tabs for selecting the larvae.
2. Left and right button to scroll through the larva tabs.
3. Change the color of the current selected larva.

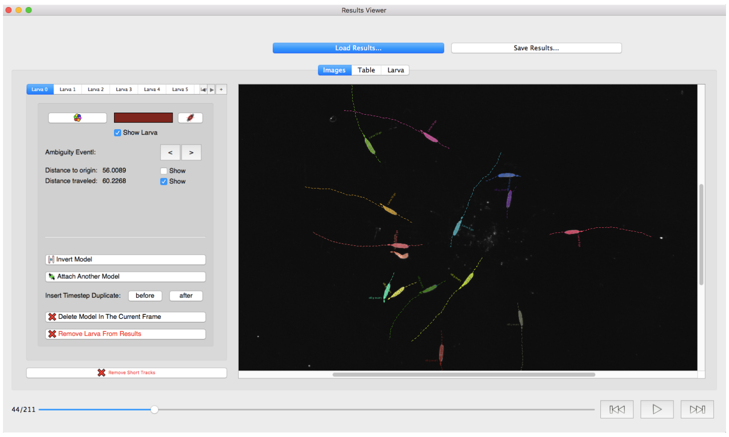

(a)

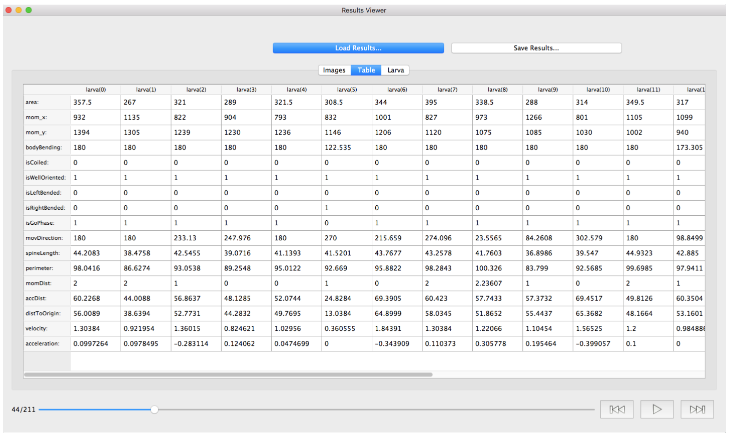

	larva(0)	larva(1)	larva(2)	larva(3)	larva(4)	larva(5)	larva(6)	larva(7)	larva(8)	larva(9)	larva(10)	larva(11)	larva(1[illegible]
area:	357.5	267	321	289	321.5	308.5	344	395	338.5	288	314	349.5	317
mom_x:	932	1135	822	904	793	832	1001	827	973	1266	801	1105	1099
mom_y:	1394	1305	1239	1230	1236	1146	1206	1120	1075	1085	1030	1002	940
bodyBending:	180	180	180	180	180	122.535	180	180	180	180	180	180	173.305
isCoiled:	0	0	0	0	0	0	0	0	0	0	0	0	0
isWellOriented:	1	1	1	1	1	1	1	1	1	1	1	1	1
isLeftBended:	0	0	0	0	0	0	0	0	0	0	0	0	0
isRightBended:	0	0	0	0	0	1	0	0	0	0	0	0	0
isGoPhase:	1	1	1	1	1	0	1	1	1	1	1	1	1
movDirection:	180	180	233.13	247.976	180	270	215.659	274.096	23.5565	84.2608	302.579	180	98.8499
spineLength:	44.2083	38.4758	42.5455	39.0716	41.1393	41.5201	43.7677	43.2578	41.7603	36.8986	39.547	44.9323	42.885
perimeter:	98.0416	86.6274	93.0538	89.2548	95.0122	92.669	95.8822	98.2843	100.326	83.799	92.5685	99.6985	97.9411
momDist:	2	2	1	0	0	1	0	2	2.23607	1	0	2	1
accDist:	60.2268	44.0088	56.8637	48.1285	52.0744	24.8284	69.3905	60.423	57.7433	57.3732	69.4517	49.8126	60.3504
distToOrigin:	56.0089	38.6394	52.7731	44.2832	49.7695	13.0384	64.8999	58.0345	51.8652	55.4437	65.3682	48.1664	53.1601
velocity:	1.30384	0.921954	1.36015	0.824621	1.02956	0.360555	1.84391	1.30384	1.22066	1.10454	1.56525	1.2	0.98488[illegible]
acceleration:	0.0997264	0.0978495	-0.283114	0.124062	0.0474699	0	-0.343909	0.110373	0.305778	0.195464	-0.399057	0.1	0

(b)

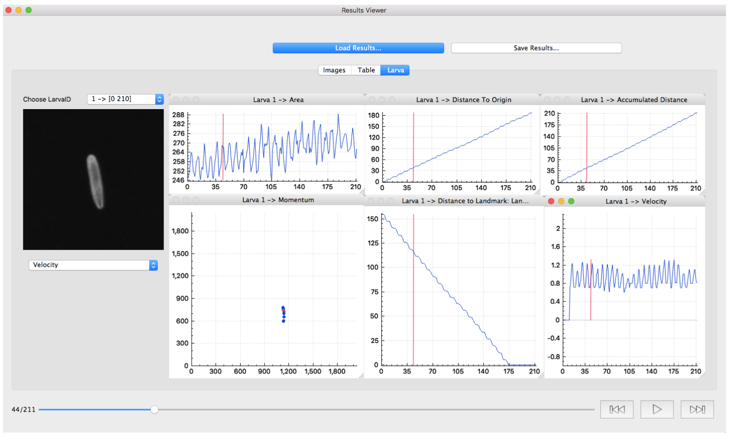

(c)

Figure 12: Sub-views of the Results Viewer. (a) Image view with color-coded plotted tracking results. (b) Table view showing current calculated features. (c) Larva view with some calculated features of a single animal.

4. Locate the larva in the image.

5. Checkbox to hide/show this larva.

6. Jump to previous (left arrow) or next (right arrow) ambiguous time point of this larva (e.g. animal is in a coiled state).

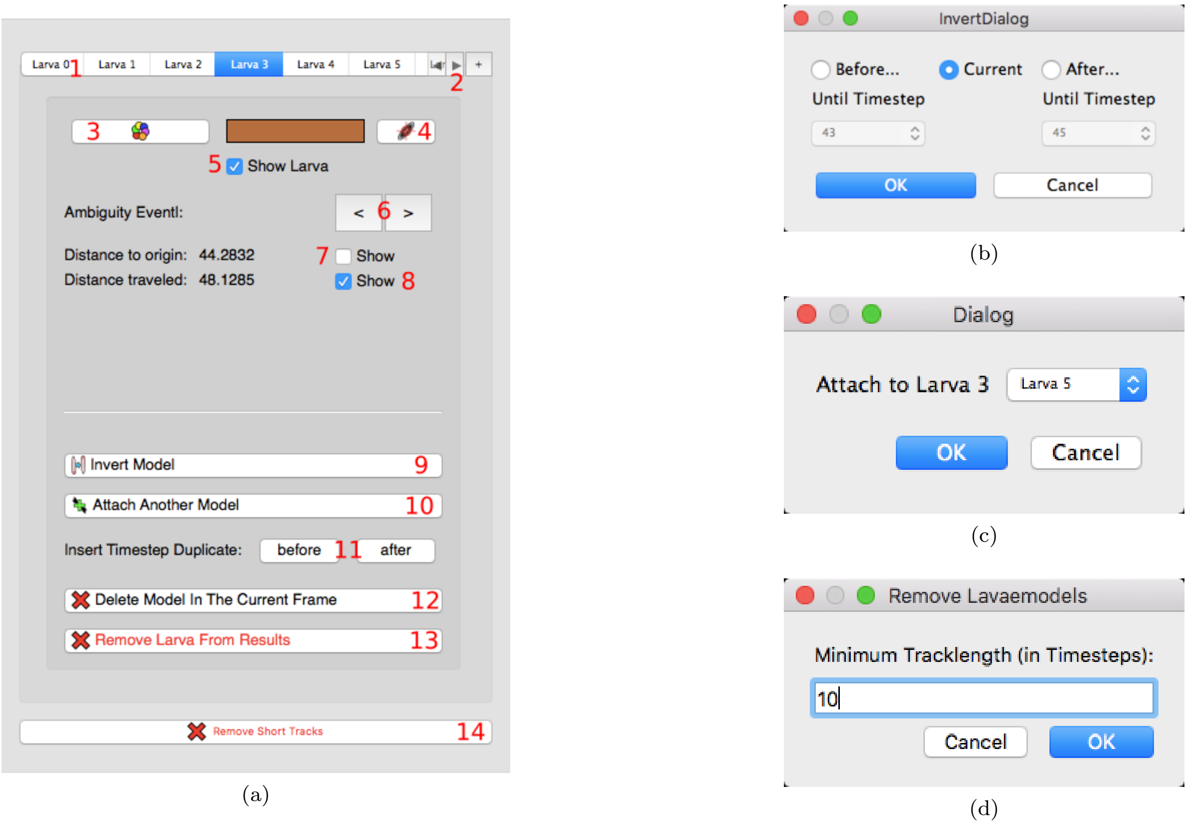

(a) (b) (c) (d)

Figure 13: Manual Tracking: (a) Edit panel (see text). (b) Invert a model of a larva. (c) Select the larva trajectory which should to be united with the current larva trajectory. (d) Dialog for removing short trajectories.

7. Distance to origin (until current time point) and checkbox for drawing this value into the image.

8. Distance traveled (until current time point) and checkbox for drawing this value into the image.

9. Invert the model (swap head and tail). The *inversion dialog* will appear (see below).

10. Attach the selected larva to another animal in order to unite the trajectories. The *attach to dialog* will appear (see below).

11. Insert a duplicate of this larva *before* or *after* the current frame.

12. Delete model in the current frame (i.e., the larva at this specific time point).

13. By clicking on *Remove Larva From Results* you will delete all measurements of the selected larva.

14. *Remove Short Tracks* can be used to remove larval trajectories which are shorter then a minimal number of frames (i.e., time points). The minimal length in frames can be set at the *remove larva* dialog (see below).

Invert Larval Model For swapping the head and tail please use the invert larval model button (9 in Figure 13a). After clicking on this button the invert larva dialog will appear (Figure 13b). It is possible to invert the model at the current frame or in all frames *Before* or *After* the frame selectable in the dropdown menu below.

Attach Another Model For uniting two trajectories use the *Attach Another Model* button. Select the model which should be attached to the current larva model (Figure 13c).

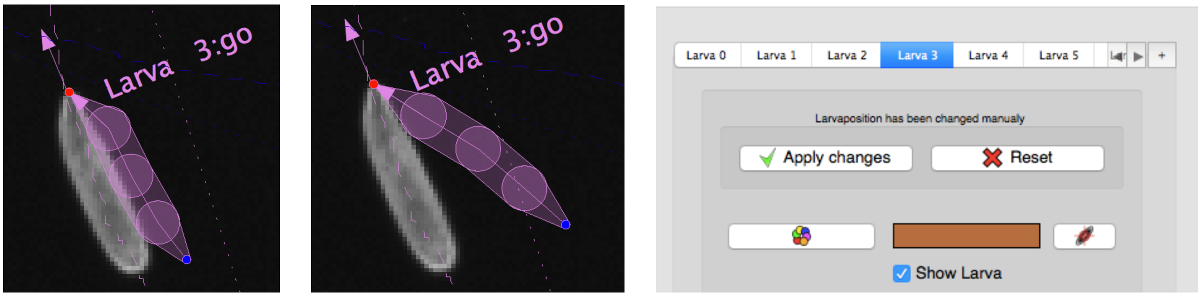

(a) (b) (c)

Figure 14: Examples of adjusted larval models. (a) Changed larval model. (b) Another changed larval model. (c) Dialog to confirm manual posture and trajectory changes.

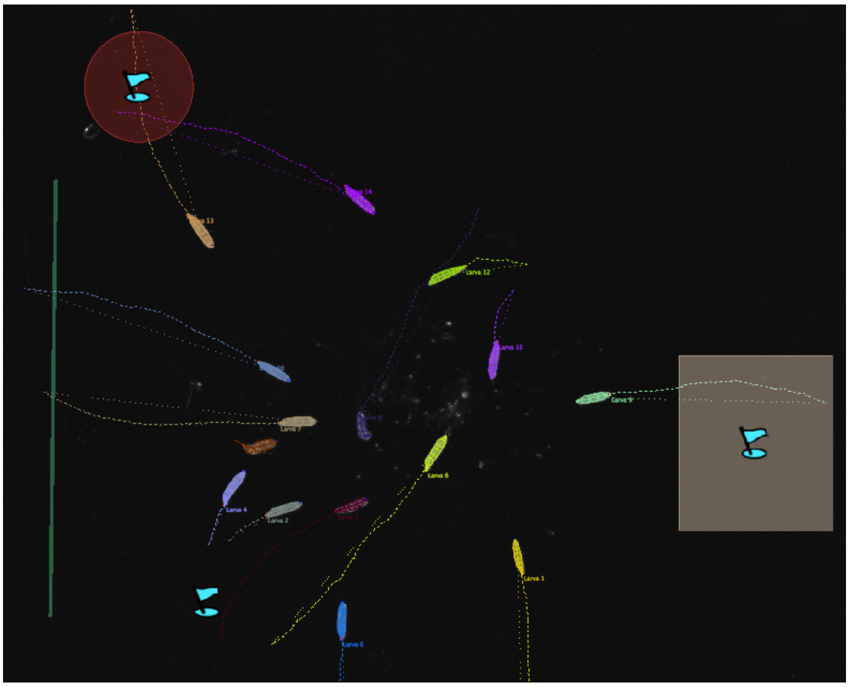

Figure 15: Several stimulus marker are placed in the *Results Viewer* image tab.

Remove Short Tracks Trajectories which are to short can be removed from the results by using the *Remove Short Tracks* button. In the following dialog, the minimal number of frames for a valid trajectory has to be entered (Figure 13d).

Adjust Single Larva You can also adjust the posture for each animal (Figure 14a and Figure 14b). First select an animal which posture you would like to adjust by selecting the larva tab of the animal (see 1 in Figure 13a). Subsequently, select the spine point you would like to change in the result image using the left mouse button. Move the mouse while holding the left mouse button to change the location of the selected spine point. You can also change the radius which is drawn around each spine point (not of the head and the tail) and which approximates the thickness of the animal by selecting the points (left mouse button) and pressing the *cmd*/*Ctrl* key while moving the mouse. All changes have to be confirmed in the edit panel to avoid unintentional changes of the measured trajectories and postures (Figure 14c).

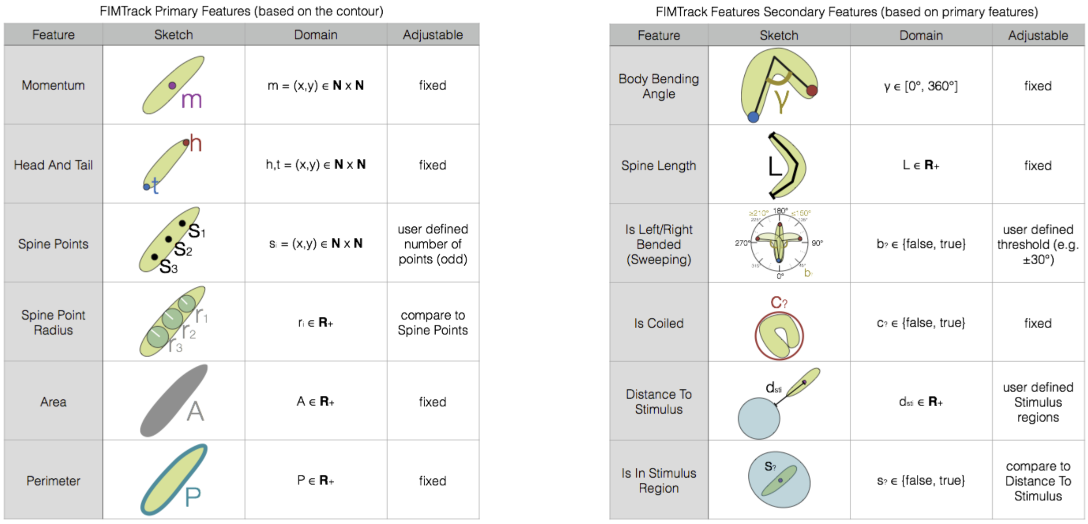

(a) (b)

Figure 16: (a) Primary features calculated by FIMTrack. (b) Secondary features calculated by FIMTrack.

2.9 Insert Stimuli

You can add different stimuli marker at the *Results Viewer* (Figure 15). A right click inside the results viewer image (at the image tab) gives you the opportunity to select one of the following stimulus marker:

- Point which is (x, y) position
- Line which is a straight line
- Rectangle which is an arbitrary sized axis-aligned 2D rectangle
- Ellipsoid which is an arbitrary sized axis-aligned 2D ellipsoid

For all markers the additional features *distance to stimulus*, *bearing angle to stimulus*, and *is in stimulus region* are calculated for each time point and animal. The size of all stimulus marker can be adjusted equivalent to the procedure described for region of interests (see Section 2.3).

2.10 Save Adjusted Results And Stimuli

To save your adjustments permanently, click on the *Save Results...* button at the top of the *Results Viewer*. At the following dialog you have to enter the (new) file names for the YAML, the CSV, and the TIFF image file.

3 Features Calculated By FIMTrack

Here is a list of all features calculated by FIMTrack. These parameters are stored in the *table.csv* and the *output.yml* file and can be separated into primary, secondary, and tertiary parameters.

3.1 Primary Features

The primary features are directly calculated from the contour of an animal.

FIMTrack Features Tertiary Features (based on primary features and time)

Feature	Sketch	Domain	Adjustable
Movement Direction	α	α ∈ [0°, 360°]	fixed
Consecutive Movement Distance	d	d ∈ $\mathbf{R}_+$	fixed
Velocity	$v = \delta \frac{d}{\Delta t}$	v ∈ $\mathbf{R}_+$ (mm/s)	user defined fps, scale and smoothing
Acceleration	$a = \frac{\Delta v}{\Delta t}$	a ∈ $\mathbf{R}_+$ (mm/s²)	compare to Velocity
Accumulated Distence	$d_{acc} = d_1 + d_2 + d_3 + \cdots$	d_{acc} ∈ $\mathbf{R}_+$	fixed
Distance To Origin	d_{org}, origin	d_{org} ∈ $\mathbf{R}_+$	fixed
Bearing Direction	β	β ∈ [0°, 360°]	compare to Distance To Stimulus
Peristalsis (Based On Area)	area, f(t), time	f(t) ∈ $\mathbf{R}_+$	fixed
Stop And Go Phase Indicator	p?, go, stop	$p_?$ ∈ {false, true}	user defined velocity and body bending angle

(a)

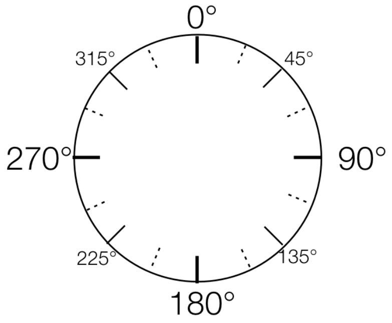

(b)

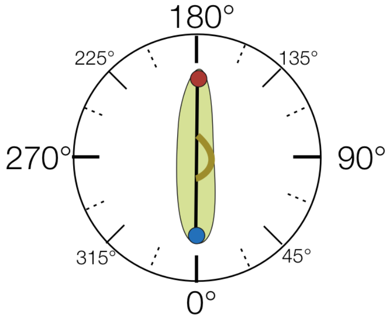

(c)

Figure 17: (a) Tertiary features calculated by FIMTrack. (b) Overall coordinate system: right is 90° and left is 270° (c) The orientation of the body bending (180° indicates no bending, 90° indicates bending to the right and 270° indicates bending to the left)

- **mom_x** and **mom_y** specifies the (x, y) position of the center of mass (momentum; in pixel coordinates).
- **head_x** and **head_y** specifies the position of the head (in pixel coordinates).
- **tail_x** and **tail_y** specifies the position of the tail (in pixel coordinates).
- **Several points** along the spine (user defined number; in pixel coordinates).
- **Radii** along the inner spine points (in pixels).
- **area** specifies the covered area (in pixels).
- **perimeter** specifies the perimeter of the contour (in pixels).

3.2 Secondary Features

Most of the secondary features are calculated based on the primary features.

- **bodyBending** specifies the body bending of an animal. Bending of 180° means no bending, values $> 180°$ indicate bending to the left and values $< 180°$ specify bending to the right (Figure 17c; in degree).
- **spineLength** specifies the length of the spine (in pixels)
- **isLeftBended** and **isRightBended** is 1 (otherwise 0) if the larva is bended to the left or to the right. This value primarily depends on the threshold which was set at the preference option *Angle Threshold*. (binary indicator).
- **isCoiled** is 1 (otherwise 0) if the larva is in a coiled state (i.e., head is touching the tail; binary indicator).
- **Distance to stimulus** is the distance to a given stimulus (in pixels).
- **In Stimulus Indicator** is 1 if the animal is inside a stimulus region, otherwise 0 (binary indicator).

3.3 Tertiary Features

The tertiary features are calculated based on the primary features and time informations.

- **movDirection** specifies the movement direction measured to the y-axes in degree. A value of 90° represents a movement to the right and a value of 270° a movement to the left (Figure 17b; in degree).
- **momDist** specifies the distance between two consecutive center of mass positions (in pixels).
- **Velocity** is the velocity of the larva (in $\frac{\text{pixels}}{\text{fps}}$)
- **Acceleration** is given in $\frac{\text{pixels}}{\text{fps}^2}$.
- **accDist** specifies the accumulated distance over time (in pixels).
- **dstToOrigin** specifies the direct linear distance to the origin of the trajectories (in pixels).
- The bearing direction specifies movement direction towards or away from a stimulus: an angle of 0° indicates that an animal moves straight towards a stimulus and a value of 180° indicates that an animals moves in the opposite direction away from the stimulus (in degree).
- The peristalsis is indirectly given by the area over time (in pixels).
- **isGoPhase** is 1 if the larva is in a go phase and 0 indicates that a larva is in a stop phases (binary indicator).

4 Contact

For more informations visit the FIM website at `fim.uni-muenster.de` or contact the FIM-Team at fim@uni-muenster.de.

References

[1] B. Risse, S. Thomas, N. Otto, T. Löpmeier, D. Valkov, X. Jiang, and C. Klämbt, "FIM, a Novel FTIR-Based Imaging Method for High Throughput Locomotion Analysis," *PloS one*, vol. 8, no. 1, p. e53963, Jan. 2013.

[2] B. Risse, X. Jiang, and C. Klämbt, "FIM: Frustrated Total Internal Reflection Based Imaging for Biomedical Applications," *ERCIM News*, vol. 95, no. Image Understanding, pp. 11–12, Oct. 2013.

[3] B. Risse, N. Otto, D. Berh, X. Jiang, and C. Klämbt, “FIM Imaging and FIMTrack: Two New Tools Allowing High-Throughput and Cost Effective Locomotion Analysis,” *The Journal of Visualized Experiments*, no. 94, p. e52207, Nov. 2014.

[4] D. Chetverikov, “A simple and efficient algorithm for detection of high curvature points in planar curves,” in *Proc. of the 10th International Conference on Computer Analysis of Images and Patterns*, Aug. 2003, pp. 746–753.

[5] H. W. Kuhn, “The Hungarian method for the assignment problem,” *Naval Research Logistics Quarterly*, vol. 2, no. 1–2, pp. 83–97, Mar. 1955.

[6] J. Munkres, “Algorithms for the assignment and transportation problems.” *Journal of the Society for Industrial and Applied Mathematics*, vol. 5, no. 1, pp. 32–38, Mar. 1957.
